# Mid-Thoracic Spinal Injuries during Horse Racing: Report of 3 Cases and Review of Causative Factors and Prevention Measurements

**DOI:** 10.1155/2013/715409

**Published:** 2013-06-11

**Authors:** Ioannis Triantafyllopoulos, Andreas Panagopoulos, George Sapkas

**Affiliations:** ^1^Orthopaedic Department, Metropolitan Hospital, Athens, Greece; ^2^Orthopaedic Department, University of Athens, Greece; ^3^Orthopaedic Department, University of Patras, Papanikolaou Street, Rio, 26504 Patras, Greece

## Abstract

We report three cases of a rare pattern of mid-thoracic spine injuries after horse racing falls and discuss possible causative factors and prevention measurements to reduce injury rates in professional riding and racing. Three patients, 2 male and 1 female with a mean age of 28 years old, underwent surgical treatment for mid-thoracic fractures after professional equestrian activities. The ASIA scale was E in one patient, B in the other one and A in the third. Multilevel posterior fusion was used in two patients and somatectomy plus fusion in the other. Follow up evaluation included changing of the ASIA scale, functional outcome and participation in equestrian activities. One patient fully recovered after surgery. Two patients remained paraplegic despite early surgical treatment and prolonged rehabilitation therapy. All patients had ended their professional equestrian career. This report analyzes possible mechanisms of injury and the pattern of mid-thoracic spine fractures after professional horse riding injuries. Despite skill improvements and continued safety education for horse riding, prophylactic measures for both the head and the spine should be refined. According to our study, additional mid-thoracic spinal protection should be added.

## 1. Introduction

In professional horse racing and riding, jockeys are amenable to a high rate of career-ending injuries mainly involving fractures of the head, shoulder or torso followed by neurological injuries to the head and/or spine [1]. 

Three cases of severe mid-thoracic spine and spinal cord injuries to professional equestrian riders are presented. The aim of this paper is: (a) to present these rare patterns of mid-thoracic spinal fractures and cord injuries, (b) to investigate the detrimental outcome of such injuries, and (c) to propose prevention and safety measurements for the horse riders. 

## 2. Materials and Methods

During the last decade we treated three rare cases of mid-thoracic spinal injuries from horse riding and racing. Neurological impairment was classified according to the American Spinal Injury Association (ASIA). Spinal fractures were classified according to the AO system [2].

**Figure 1:**
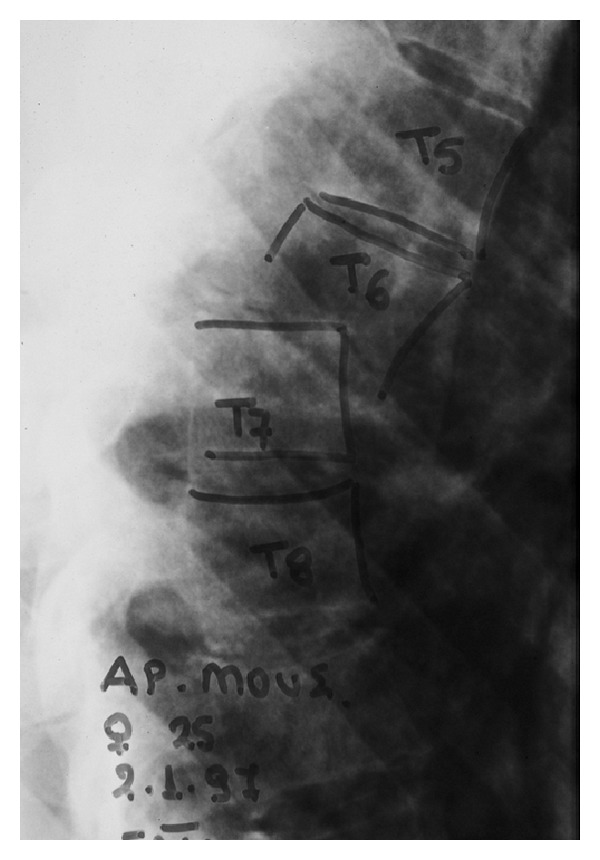
Compression fracture T5 and T7 and burst fracture T6.

*Patient 1* was a 26 years old top athletic horse rider lady, who sustained a complex mid-thoracic injury involved compression fractures of T5 and T7 (AO type A3.1.1), a burst T6 fracture (AO type B1.2.3) and T6 on T7 anterior dislocation (Figure 1). Her neurological status on presentation was ASIA-E. Associated injuries included left 6th to 8th rib fractures and haemothorax. She underwent T6 and partial T7 laminectomy, removal of T6 bony fragments from canal followed by T3 to T9 posterior fusion using the Isola spinal instrumentation system (*DePuy Inc.*).

**Figure 2:**
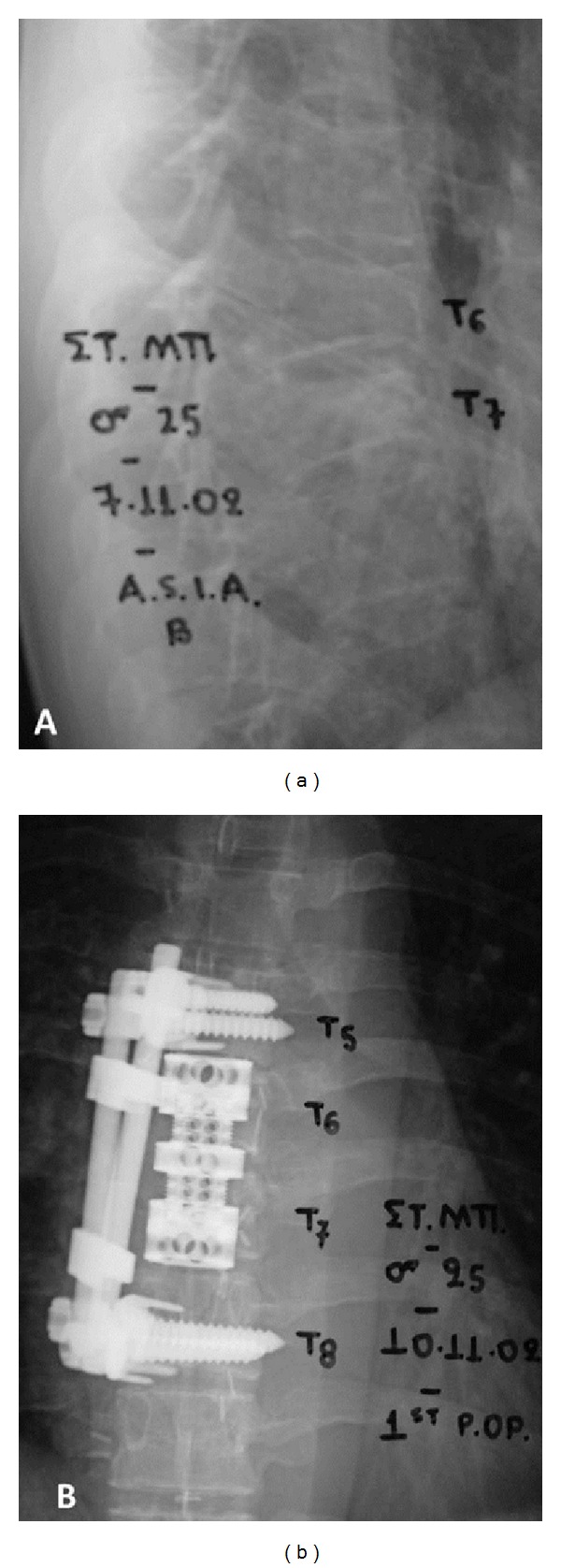
(a) Burst fracture T7 and old compression fracture T6 conservatively treated. (b) T5–T8 anterior fusion with Kaneda spinal instrumentation (*DePuy Inc*.) and T6-T7 somatectomy and replacement with VBR (vertebral body replacement) system (*Penny-Ultra Inc.*).

*Patient 2* was a 25 years old male professional horse racing jockey who sustained a burst T7 fracture (AO type A2.2) without any other concomitant injury (Figure 2(a)). His neurological status on presentation was ASIA B. There was also an old compression fracture on T6 treated conservatively. He underwent decompression, T6 and T7 somatectomy with VBR (vertebral body replacement) system (*Penny-Ultra Inc.*) and T5-T8 anterior fusion using the Kaneda spinal instrumentation system (*DePuy Inc*.) (Figure 2(b)).

**Figure 3:**
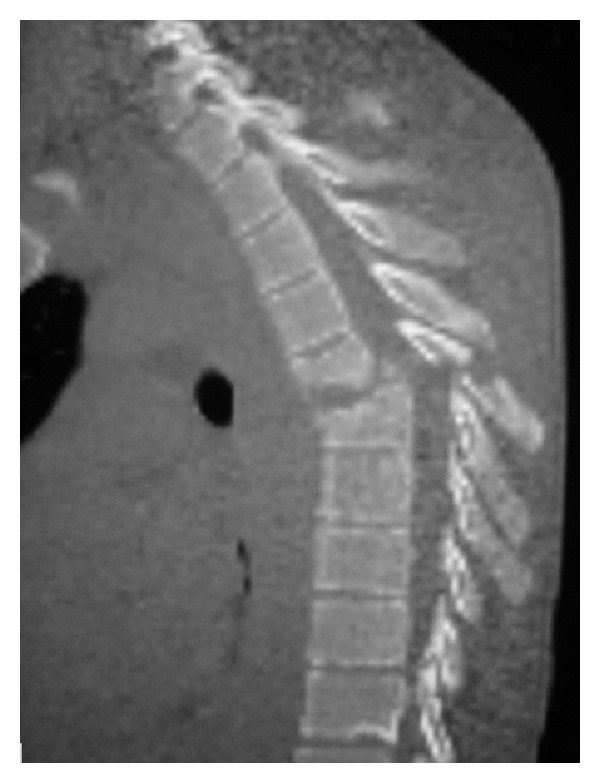
Fracture and bilateral dislocation T6 on T7.

*Patient 3* was a 32 years old male professional horse racing jockey who sustained fractures of T6 (AO type A1.3) and T7 (AO type B2.1) and bilateral dislocation T6 on T7 (Figure 3). His neurologial status on presentation was ASIA-A. Associated injuries included multiple rib fractures (left 1st–4th rib and right 1st rib) and bilateral haemothorax. He underwent T6 and T7 partial laminectomy, followed by T4–T10 posterior fusion using the Legacy spinal instrumentation system *(Medtronic Sofamore Danec Inc.)*.

## 3. Results

 All three patients had received special rehabilitation therapy. Patient-1 did not develop any neurological deficit. Instrumentation was removed 9 months after initial surgery. Despite her fully recovery she decided to stop participating in any equestrian activities. Patient-2 was transferred postoperatively to a special rehabilitation centre but never showed any neurological improvement. Patient-3 was also transferred postoperatively to a special rehabilitation centre but only the sensory function was slightly improved.

## 4. Discussion

The principle finding of the present report is that there is a rare pattern of mid-thoracic horse riding injuries which can lead to severe functional and neurological impairment. Equestrianism accounts for the largest number of hospital admission days when compared to all other sports. The incidence of serious injuries and fatal accidents in both genders and at any age group is very high and surprisingly higher than motorized sports [3]. Recent surveys have shown that 20% of injured riders attending hospital are admitted and approximately 60% of these have head injuries [4]. There are certain dangers that are inherent to riding on a horse: a full grown animal can weigh over 500 kg and can kick with a force of 1.8 times its bodyweight. At full gallop, race horses develop speeds of up to 65 km/h (40 mph). When mounted, the rider's head is poised up to 4 meters (13 feet) from the ground. Considering that riders trying to control a less intelligent animal with different and unpredictable reactions, there is always the potential risk of unexpected behaviour from either the horse or the rider. In addition, accidents may occur from reasons unrelated to ridding, such as car collisions when riding on the road. 

The estimated incidence for farming and equestrian injuries in our country [5] has been recently estimated as 21 per 100,000 person-years, but it was 160 times higher for horse-racing personnel. Spine injuries accounted for approximately 7% in this subgroup of patients. Several other studies confirmed that 7–10% of all riders requiring hospital admission will have a spinal injury [4, 6]. More recently Silver [4] and Hasler et al. [7] reported higher incidence of spinal fractures (17% and 14% in respect). Although numerically few, catastrophic injuries to the spine and spinal cord from horse riding giving rise to paralysis have attracted considerable attention. First aid and stabilization at the site of injury, immediate transfer to a special unit, management of the fracture and spinal cord injury and long term rehabilitation are the same as in any other spinal injury but specific questions need to be addressed regarding the mechanism of injury and the type of equestrian activity more amenable to spinal injuries, the part of the spine more often damaged, the commonest fractured level and how could horse riding can be made safer.

### 4.1. Mechanism of Injury & Type of Equestrian Activity

Jumping and point to pointing are the most dangerous equestrian activities compared to flat racing or social riding [8]. This is in accordance with the only three cases of spinal injuries caused during horse racing we treated the last decade. 

In Australia, injury rates were found to be especially high among event riders and in the USA cross country schooling accounted for 22.5% of accidents at pony clubs [4]. In the UK the Jockey Club reports that point to pointing is more dangerous than any other horse riding activity, carrying the risk of a fall in one in seven rides, injury one in 42 rides and one in 4.5 falls [8]. Chitnavis et al. [9] state that the majority of the injured were riders (78%) of whom the majority fell from their mounts (83%). Some were crushed as their horse fell (14%) and others struck obstacles whilst mounted (5%) or were injured by entrapment of the reins (5%). In horse racing it is virtually impossible to correlate specific pattern of falls with certain spinal injuries as several other factors play an important role to the likehood of serious injury such as the proximity of other horses which can lead to injuries not only because of the fall of the rider but also because of the fall of a horse onto the prone rider as well as the unpredictable reactions of both jockeys and horses in high speed rates during professional horse racing [10]. Greater skill and experience in equestrian sports is a contentious hypothesis of greater safety as the demands increase at the higher competitive levels [11]. Hasler et al. [7] in a retrospective data analysis with a case-control study in 365 injured riders reported that older age, male gender and the possession of a diploma in horse riding can be protective factors in equestrian injuries. 

### 4.2. Site of Injury

In an overall review of horse riding injuries [12], head injuries outnumber spinal injuries at about 5 : 1 this would indicate that the force required to cause a head injury is rather less than that required fracturing the spine. This information indicates the importance of protecting helmets [13]. In horse racing (cross country position), the jockey keeps the head forward and would be more likely to sustain a cervical injury accompanied inevitably by a head injury. However, in all three cases reported in this paper, jockeys sustained a thoracic spinal fracture instead of a cervical spine and/or head injury. In horse riding (classical style position), the rider's head is held high. As such, the rider would be likely to fall onto the buttocks. This mechanism of fall predisposes to thoracic and lumbar spinal injuries. Admissions to spine units for horse riding accidents are far more for lumbar and thoracic injuries than for cervical injuries in contrast to all other sporting activities [14]. Again, this information indicates the importance of special spine jackets for the prevention of spinal injuries. Hessler et al. [15] conducted a questionnaire survey on 30 horse riders suffering from spine injuries and found that 7 (23%) wore a safety vest at the time of injury. He concluded that the spine can get damaged even though protective body gear is worn, especially when the energy impact is too high. 

### 4.3. Spine Fracture Level

In horse riding the thoraco-lumbar junction seems to be more vulnerable [4]. Thoraco-lumbar fractures are almost unique to equestrian activities. Siebenga et al. [6] reported a 78% incidence of thoracolumbar fractures (T11-L2) in 36 spine fractures among 32 patients; only 3 patients sustained a mid-thoracic fracture. In recreational horse riding, the usual mechanism of such an injury is when riders are thrown against obstacles such as tree branches, which unseat riders and push them backwards. In event horse riding, the usual mechanism of thoraco-lumbar injury is a fall off the horse while jumping. However, all our three cases sustained a mid-thoracic fracture at the levels of T5–T8 which is not a very common site of spinal injury. Furthermore, the incidence of acute spinal cord injuries during a horse racing accident is not high [4, 14]; nonetheless, two out of three of our patients sustained a spinal cord injury.

## 5. Conclusion

In view of the many different injuries described in literature, one can say that there is no activity with horses which is entirely without risk of injury. Equestrian injury prevention initiatives should further define groups at risk and focus on safe riding practices, proper horse handling, and educating riders in horse behavior. One suggestion is to teach horse riders falling techniques as used in martial arts sports and parajumping Mid-thoracic spine injuries should be added to the list of equestrian injuries and measurements to improve spinal safety should also include this anatomic site of mid-thoracic spine. 

## References

[1] Balendra G, Turner M, McCrory P (2008). Career-ending injuries to professional jockeys in British horse racing (1991–2005). *British Journal of Sports Medicine*.

[2] Magerl F, Aebi M, Gertzbein SD, Harms J, Nazarian S (1994). A comprehensive classification of thoracic and lumbar injuries. *European Spine Journal*.

[3] Ball JE, Ball CG, Mulloy RH, Datta I, Kirkpatrick AW (2009). Ten years of major equestrian injury: are we addressing functional outcomes?. *Journal of Trauma Management & Outcomes*.

[4] Silver JR (2002). Spinal injuries resulting from horse riding accidents. *Spinal Cord*.

[5] Petridou E, Kedikoglou S, Belechri M, Ntouvelis E, Dessypris N, Trichopoulos D (2004). The mosaic of equestrian-related injuries in Greece. *Journal of Trauma*.

[6] Siebenga J, Segers MJM, Elzinga MJ, Bakker FC, Haarman HJTM, Patka P (2006). Spine fractures caused by horse riding. *European Spine Journal*.

[7] Hasler RM, Gyssler L, Benneker L (2011). Protective and risk factors in amateur equestrians and description of injury patterns: a retrospective data analysis and a case—control survey. *Journal of Trauma Management and Outcomes*.

[8] Press JM, Davis PD, Wiesner SL, Heinemann A, Semik P, Addison RG (1995). The national jockey injury study: an analysis of injuries to professional horse-racing jockeys. *Clinical Journal of Sport Medicine*.

[9] Chitnavis JP, Gibbons CLMH, Hirigoyen M, Lloyd Parry J, Simpson AHRW (1996). Accidents with horses: what has changed in 20 years?. *Injury*.

[10] Edixhoven P, Sinha SC, Dandy DJ (1981). Horse injuries. *Injury*.

[11] Whitlock MR, Whitlock J, Johnston B (1987). Equestrian injuries: a comparison of professional and amateur injuries in Berkshire. *British Journal of Sports Medicine*.

[12] Hamilton MG, Tranmer BI (1993). Nervous system injuries in horseback-riding accidents. *Journal of Trauma*.

[13] Lim J, Puttaswamy V, Gizzi M, Christie L, Croker W, Crowe P (2003). Pattern of equestrian injuries presenting to a Sydney teaching hospital. *ANZ Journal of Surgery*.

[14] Silver JR (1993). Spinal injuries in sports in the UK. *British Journal of Sports Medicine*.

[15] Hessler C, Namislo V, Kammler G, Lockemann U, Püschel K, Meenen NM (2011). Spine injuries due to horse riding accidents—an analysis of 30 cases. *Sportverletzung-Sportschaden*.

